# Radiative Defects in Chloride-Activated CdSe Thin Films

**DOI:** 10.1021/acsenergylett.5c03191

**Published:** 2026-01-26

**Authors:** Abasi Abudulimu, Xiaoming Wang, Tyler Brau, Jaroslav Kuliček, Scott L. Wenner, Adam B. Phillips, Ebin Bastola, Manoj K. Jamarkattel, Vijay C. Karade, Kiran Lamichhane, Aparajita Dixit, Bohuslav Rezek, Yanfa Yan, Michael J. Heben, Randy J. Ellingson

**Affiliations:** † Wright Center for Photovoltaics Innovation and Commercialization (PVIC), Department of Physics and Astronomy, 7923 The University of Toledo, Toledo, Ohio 43606 United States; ‡ Faculty of Electrical Engineering, 48220 Czech Technical University in Prague, Technická 2, 16627 Prague, Czechia

## Abstract

Defect recombination limits wide-gap Se-based chalcogenide devices, yet how chloride activation reshapes radiative pathways remains unclear. Here we show that a 40 min CdCl_2_ anneal converts evaporated CdSe from porous nanograins into dense micrometer-scale polycrystals and sharpens the optical band edge, reducing the Urbach energy from 85 to 17 meV at 300 K. Combining temperature- and fluence-dependent photoluminescence (PL), time-resolved PL, hyperspectral mapping, and hybrid-DFT, we resolve three emissive channels and identify their mechanisms. The near-edge band is excitonic at low temperature and evolves into free-carrier emission at elevated temperature. A sub-gap band at *E*_*g*_ −0.45 eV requires above-gap carriers and thermally quenches with a 0.16 eV activation energy. A broad ∼1.05 eV infrared band is excited by above- and below-gap photons and retains microsecond lifetimes at room temperature; patial mapping links it to edge-rich microstructure. Calculations suggest selenium-vacancy and cadmium-vacancy–chlorine complexes, pointing to routes to suppress defect-related losses in wide-gap chalcogenide devices.

## Graphical abstract

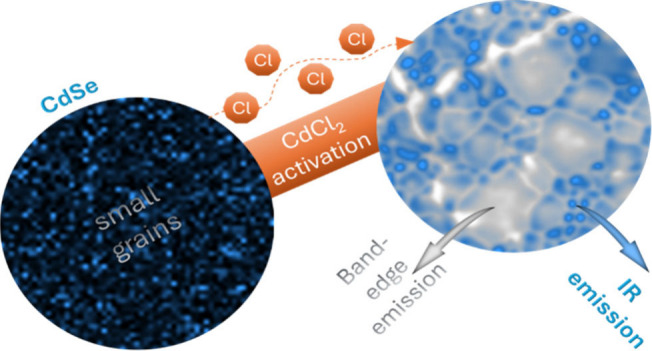

CdSe’s direct gap (*E*_*g*_ = 1.7–1.75 eV) underpins photodetectors,^1^ blue-green emitters,^2^ wide-gap absorbers for single and multijunction solar cells,^3–7^ and the Se-rich front region in Cd­(Se,Te) photovoltaics.^8,9^ Defects—not band structure—now set the ceiling for CdSe-based optoelectronics.^1,10–13^ Even the “simple” photoluminescence (PL) of pristine CdSe films and single crystals remains disputed, with conflicting assignments of near-band-edge and sub-gap bands,^14–17^ and little consensus has been reached on how temperature, injection, and microstructure couple to radiative pathways. Yet chloride activation that enables device-grade microstructure also rewrites the defect landscape,^4,9,18–20^ motivating a systematic re-examination.

Here we resolve that picture for CdCl_2_-activated CdSe thin films by combining temperature- and fluence-dependent PL, time-resolved PL (TRPL), hyperspectral confocal mapping, and first-principles defect calculations (Hybrid-DFT). After confirming that a 40 min CdCl_2_ anneal converts porous ≤ 100 nm grains into dense μm-scale polycrystals and sharpens the absorption edge (Urbach energy decreasing from 85 to 17 meV), we isolate three emissive channels: (i) a near-edge manifold that tracks *E*_*g*_(*T*); (ii) a sub-gap band at ∼*E*_*g*_ −0.45 eV that red-shifts with temperature and is spectrally stable with injection, with a thermal quenching energy *E*_*a*_ ≈ 0.16 eV; and (iii) a deep infrared band near ∼1.05 eV that blue-shifts above ∼100 K and at high injection, with *E*_*a*_ ≈ 0.038 eV. Kinetically, TRPL shows multiexponential decays spanning ∼1–800 ns (near-edge) and **∼**50–1600 ns (∼*E*_*g*_ −0.45 eV), consistent with band-edge radiative paths coupled to trapping/detrapping,^21–23^ whereas the ∼1.05 eV band is single-exponential and long-lived (≈3.4 μs at 10 K; ≈2.7 μs at 300 K), indicating a dominant deep-center radiative channel with weak temperature dependence.^16–18^ Hyperspectral maps tie these channels to microstructure, revealing grain-interior versus boundary contrasts in band-edge intensity, center energy, and line width, and pinpointing where long-wavelength defect emission gains weight. Hybrid-DFT narrows the chemically plausible defect centers to a small set consistent with the measured energetics and line-shapes.

By quantitatively separating radiative channels across 0.78–1.85 eV, extracting phonon coupling and activation barriers, and linking spectra to real microstructure and specific defects, we provide new insights and a coherent working assignment for CdSe photoluminescence characteristics that can guide future research. The framework directly informs Cd­(Se,Te) junction/device engineering,^8–13,24^ sets targets for suppressing defect recombination in single junction indoor PV and tandem top cells, and suggests routes to tunable deep-IR emission in detectors.

## Structural Evolution under CdCl_2_ Activation

Figure 1a, as-deposited CdSe, shows dense nanoscale domains (≤150 nm) with no discernible crystal facets. CdCl_2_ treatment converts the film into a network of polygonal grains routinely exceeding 1 μm (Figure 1b), with sharp triple junctions and straight boundaries pointing to solid-state recrystallization.^4,18–20^ Cross-sectional SEM (SI, Figure S1) confirms that this transformation spans the full film thickness, with no (diffusion-induced) gaps or secondary layers detectable at the interface.^20^ X-ray diffraction (XRD) (Figure 1e) identifies hexagonal CdSe in both films consistent with literature. The 24–27° group corresponds to (100)/(002)/(101), with higher-angle peaks from (102), (110), (103), and (112).^20^ After CdCl_2_, peaks sharpen and intensify, consistent with SEM-observed recrystallization. Extracted lattice constants are a = 4.33 Å and c = 7.02 Å in line with literature.^25^

**Figure 1:**
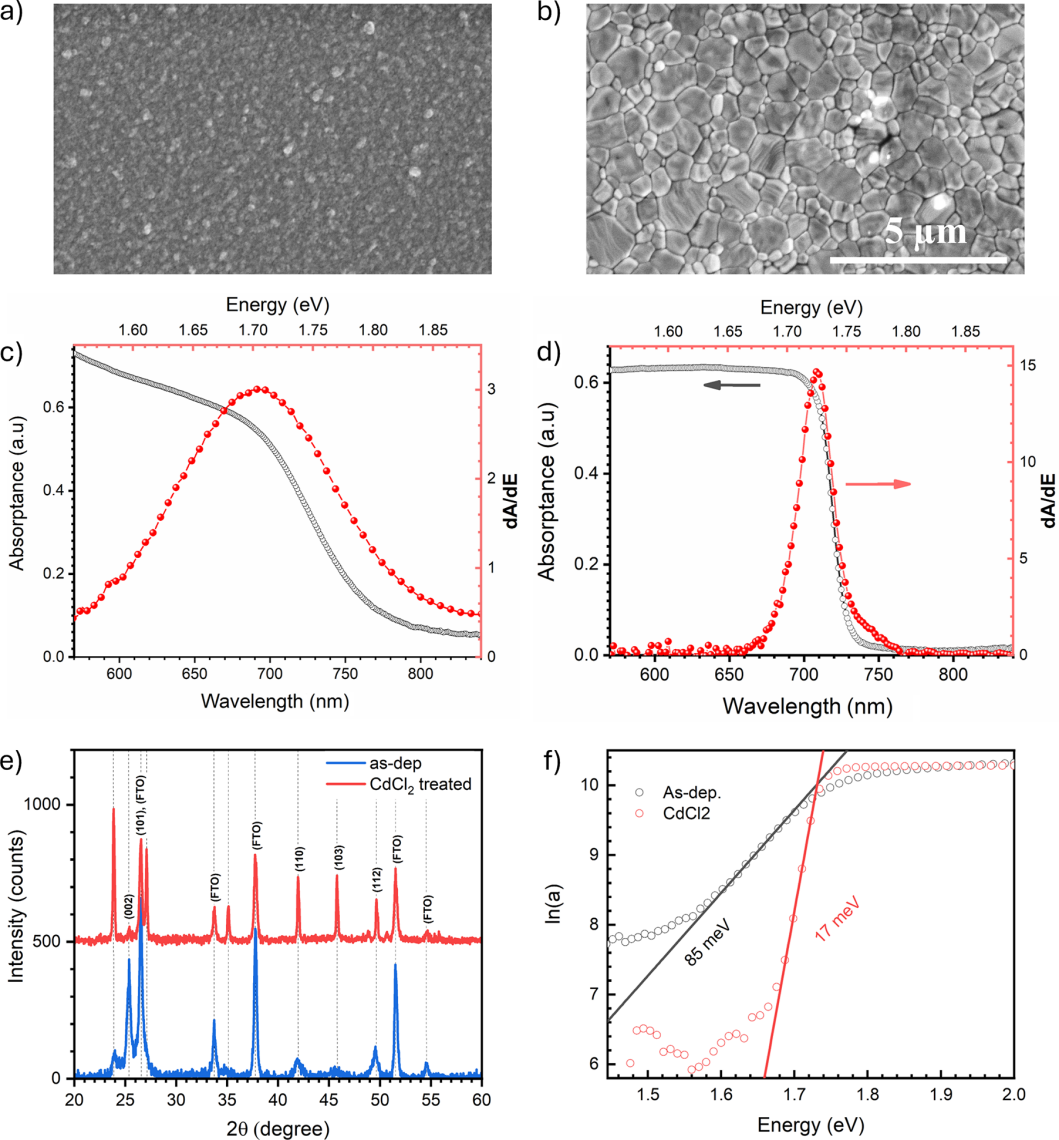
Plan-view SEM of (a) as-deposited and (b) CdCl_2_-treated CdSe thin films (scale bar: 5 μm). (c,d) Room-temperature absorptance (gray, left axis) and its energy derivative dA/dE (red, right axis) for the as-deposited and CdCl_2_-treated films, respectively; the derivative is used to locate E_g_. (e) XRD patterns of as-deposited and CdCl_2_-treated films; reflections from the FTO substrate are indicated. (f) Urbach analysis: ln­(A) vs photon energy with linear fits in the exponential tail region, yielding E_U_ ≈ 85 meV (as-deposited) and E_U_ ≈ 17 meV (CdCl_2_-treated).

Figure 1c-d display the room-temperature absorptance (gray, left axis) and its energy derivative (red, right axis) for the as-deposited and CdCl_2_-treated films. The treated film shows a much sharper inflection near 710 nm; the derivative shows a narrow peak at E_g_ ≈ 1.73 eV, whereas the as-deposited film exhibits a broader feature near 1.70 eV. Urbach analysis (Figure 1f) yields E_U_ ≈ 85 meV for the as-deposited and E_U_ ≈ 17 meV for the CdCl_2_ -treated film (see SI.5 for details). The large reduction in band-tailing, together with the grain growth and peak sharpening in XRD, indicates that chloride-induced recrystallization markedly suppresses band-edge disorder and band-tail states.^18,20^

## Temperature-Dependent Photoluminescence with above and below Gap Photoexcitation

Figure 2 summarizes how the CdCl_2_-treated film emits from 15–300 K under above-gap (532 nm) and below-gap (785 nm) excitation. **Under above-gap pumping**, three bands are resolved at 15 K (Figure 2a–b): a narrow near-band-edge line ∼681 nm (Peak 1), a broader sub-gap band ∼910 nm (Peak 2), and a deep-infrared band ∼1180 nm (Peak 3). With increasing temperature, Peak 1 weakens and red-shifts to 720 nm by 300 K; Peak 2 rises slightly to ∼120 K, then quenches becoming indistinct above ∼260 K, and it also red-shifts; Peak 3 decreases only weakly but blue-shifts. A closer look at Peak 1 below ∼60 K reveals at least three narrow components near ∼681, 690, and 703 nm (SI, Figure S2), consistent with an excitonic manifold (free and bound).^14,15^ These lines broaden and merge into a single near-edge band above ∼80–100 K as excitons ionize and phonon scattering grows. At low temperature, the near-edge emission (Peak 1) resolves into multiple narrow excitonic components (SI Figure S2), which should be distinguished from the three main emissive bands (Peaks 1–3) discussed throughout this work.

**Figure 2:**
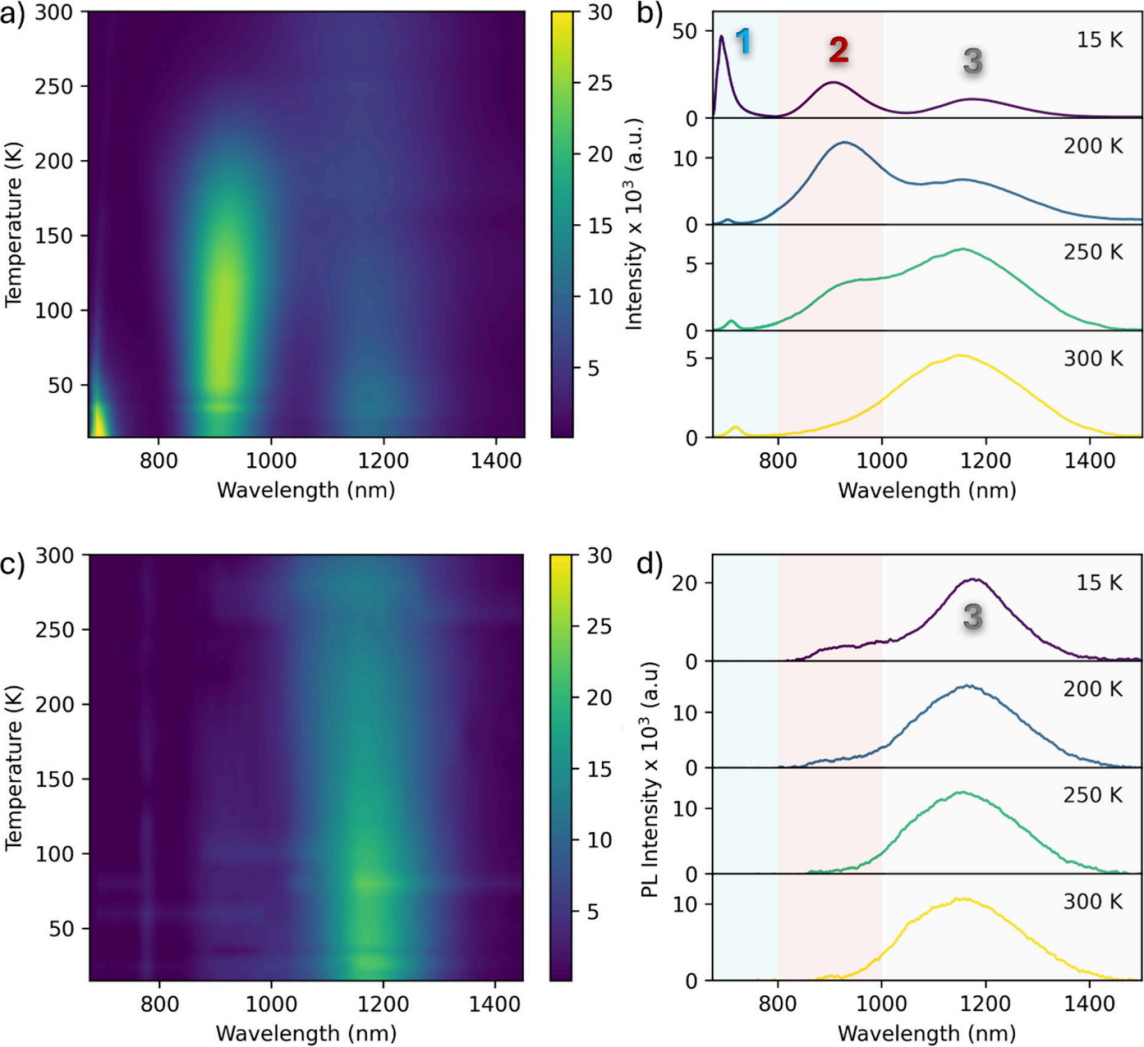
Temperature-dependent photoluminescence of CdCl_2_-treated CdSe. (a) False-color contour map of PL intensity recorded under above-bandgap 532 nm excitation, (b) Representative spectra extracted from panel (a) at 15, 200, 250, and 300 K, (c) Contour map obtained under sub-band gap 785 nm excitation and (d) the selected spectra at four different temperatures. Note, under 785 nm excitation the spectrum is dominated by Peak 3, and Peak 2 is not observed within the detection limit. The weak (featureless tail) signal between 850–1000 nm at 15 K arises from background contributions and the high-energy tail of Peak 3.

**Under below-gap excitation** (Figure 2c–d), Peak 1 and Peak 2 are absent, but Peak 3 is present with essentially the same spectral shape and temperature trend observed under 532 nm pumping. This confirms that Peaks 1 and 2 require carriers promoted across the bandgap but not Peak 3, which behaves as emission from a compact, strongly localized deep center;^16–18^ below we provide a specific working microscopic assignment supported by hybrid-DFT. For comparison, the as-deposited film shows only a broad, weak ∼1400 nm band (SI, Figure S3). The qualitative trends observed in Figure 2 are quantified in Figure 3.

**Figure 3:**
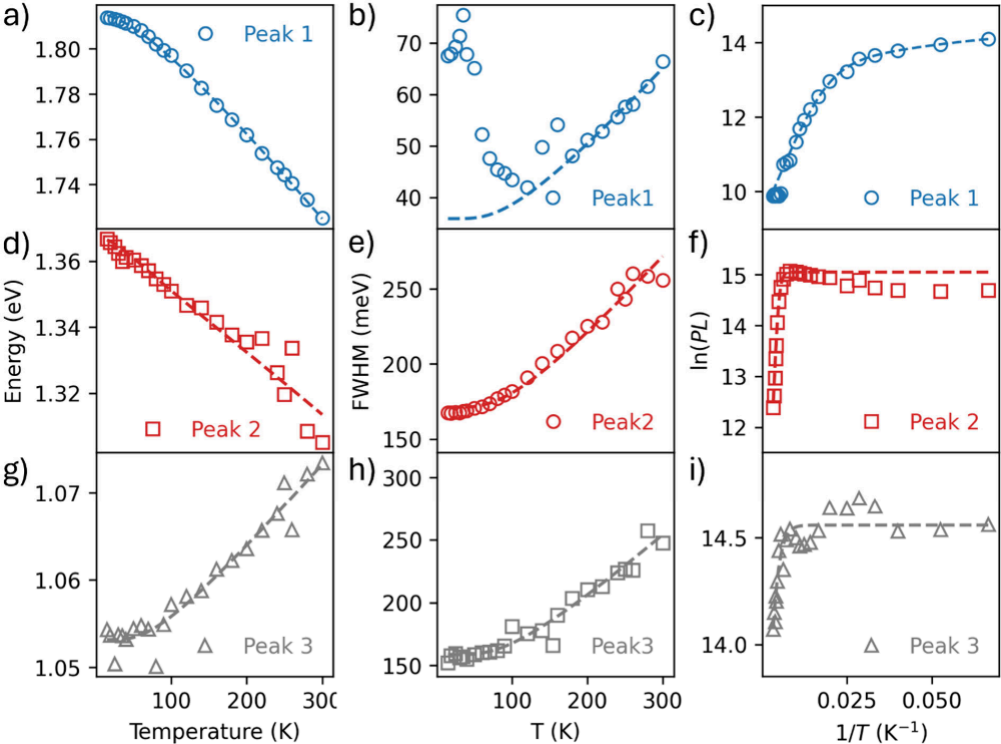
Quantitative analysis of the temperature dependence of the three emissive bands identified in Figure 2 (532 nm excitation). (a, d, g) Extracted center energies of three emission peaks: (a) Peak 1 (∼690 nm), (d) Peak 2 (∼910 nm), and (g) Peak 3 (∼1180 nm)—as a function of temperature from 15 to 300 K, showing typical redshift (Peak 1 and 2) and blueshift (Peak 3) behavior. (b, e, h) Full width at half-maximum (fwhm) of each peak versus temperature. (c, f, i) Natural logarithm of integrated PL intensity plotted against inverse temperature (1/T), revealing the thermal quenching behavior of each emission band. Dashed lines are the fits used to extract activation energies and line width broadening parameters via eqs 1–6).

Figure 3 compiles the temperature dependences of the peak center (a, d, g), integrated spectral weight (b, e, h), and line width (fwhm; c, f, (i) on common axes so their shifts, quenching onsets, and broadening can be compared directly.

**Peak 1** (near-edge). The center energy follows the band gap shrinkage with temperature and is fitted with the Varshni relation,^26^

1 $$E_{g}\left(T\right)=E_{g}\left(0\right)-\frac{\alpha T^{2}}{\beta +T}$$

where *E*_*g*_(0) is the bandgap energy at 0 K, and α and β are material-specific phenomenological parameters describing the temperature dependence of the band gap within the Varshni formalism.^26^ Physically, this temperature dependence arises from the combined effects of electron–phonon coupling and lattice dilatation (thermal expansion of the lattice with increasing temperature), which reduces orbital overlap and governs bandgap renormalization.^27,28^ The fit yields *E*_*g*_(0) = 1.816 ± 0.003 eV, α ≈ (4.1 ± 0.1) × 10^–4^ eV/K, and β ≈ 111 ± 10 K; these values fall within the range reported for II–VI semiconductors.^27,28^

Below 80 K, Peak 1 comprises overlapping excitonic sub-bands (SI, Figure S2); above ∼120 K it is a single near-edge line. We therefore quantify broadening only for *T* ≥ 120 K using^29^

2 $$FWHM\left(T\right)=FWHM_{0}+\gamma_{ac}T+\gamma_{\mathrm{LO}}N_{\mathrm{LO}}\left(T\right),N_{\mathrm{LO}}\left(T\right)=\frac{1}{\exp\left(E_{\mathrm{LO}}/k_{B}T\right)-1}$$

where *FWHM*_0_ is the inhomogeneous width at 0 K, *y*_*ac*_ and *y*_LO_ quantify acoustic and polar-LO phonon broadening, respectively, *E*_LO_ is the LO-phonon energy (25 meV^30^), and *k*_B_ is the Boltzmann constant. The fit yields *y*_*ac*_ ≈ 0 and *FWHM*_0_ = 35.9 meV, and *y*_LO_ = 47.5 meV.

The spectral weight requires a double-Arrhenius form,^17,31^

3 $$I\left(T\right)=\frac{I_{0}}{1+Ae^{-E_{a}/k_{B}T}+Be^{-E_{b}/k_{B}T}}$$

where *I*_*0*_ is the low-temperature radiative limit, and *A*, *B* are dimensionless prefactors that weigh two nonradiative channels with activation energies *E*_*a*_ and *E*_*b*_. The fit yields *E*_*a*_ = 19.3 meV and *E*_*b*_ = 2.6 meV. Restricting the fit to *T* > 120 K reduces to a single Arrhenius with *E*_*a*_ = 22.1 meV (SI, Figure S4), consistent with the exciton ionization setting the main quench once the excitonic manifold collapses.^14,15,21,22^

**Peak 2 (** *E*_*g*_ **– 0.45 eV)**. The center energy red-shifts with *T* and closely tracks *E*_*g*_ *(T)*. We model it as a fixed offset (Δ_2_) from the gap with a small LO correction:^26,31^

4 $$E_{2}\left(T\right)=E_{g}\left(T\right)-\Delta_{2}+\gamma_{LO}N_{LO}\left(T\right)$$

which yields Δ_2_ = 447 ± 1 meV and *y*_LO_ = 50 ± 6 meV.

The line width grows from 0.17 eV (15 K) to 0.27 eV (300 K). Equation 2 over the full range gives fwhm_0_ = 163 ± 3 meV, *y*_*ac*_ = 0.166 ± 0.05 meV/K, and *y*_*LO*_ = 91 ± 22 meV. A two-parameter variant with acoustic *y*_*ac*_ = 0 returns fwhm_0_ = 171 ± 2 meV and *y*_*LO*_ = 164 ± 6 meV with indistinguishable residuals, indicating covariance between the temperature-dependent terms.

Fitting the spectral weight *I*_2_(*T*) to a single-Arrhenius (eq 3 with B = 0) gives *E*_*a2*_ = 162.3 ± 6 meV. The initial rise below 120 K—visible as a turnover at 1/*T*≈ 8.3 × 10^–3^ K^–1^—is consistent with thermal feeding from the near-band-edge manifold;^14,15,31^ above ∼120 K a 0.16 eV nonradiative pathway dominates.

**Peak 3 (1.05 eV)**. Unlike Peaks 1–2, the center energy is flat to ∼100 K and then increases almost linearly, blue-shifting by ∼20 meV by 300 K. A Bose–Einstein model^32^ captures this “hinge”,

5 $$E_{3}\left(T\right)=E_{3}\left(0\right)+n\left(T\right)\hbar \omega ,n\left(T\right)=\frac{1}{\exp\left(\hbar \omega /k_{B}T\right)-1}$$

which yields *E*_*3*_ *(*0) = 1.053 ± 0.002 eV and a local vibrational energy *ℏω* = 19 ± 2 meV. The crossover *T* ≈ *ℏω/(2k*_B_ *)* ≈ 110 K matches the onset of the slope. Conventional DAP expressions^33^ that combine *E*_*g*_(*T*) with a Coulomb term tend to overcurve over 15–300 K (SI, Figure S5).

The line width is nearly constant to ∼100 K then rises linearly, mainly on the high-energy side (SI, Figure S6). Two models reproduce this. (i) eq 2 with *y*_ac_ = 0 gives *FWHM*_0_ = 170 ± 2 meV and *y*_LO_ = 148 ± 6 meV. (ii) A Huang–Rhys form,^34^

6 $$FWHM\left(T\right)=A\sqrt{\coth\left(\frac{\hbar \omega }{2k_{B}T}\right)}$$

yields *A* = 168 ± 2 meV, coupling factor *S* = 7 ± 0.1 (as *A ≈ C* $\sqrt{S}$ *ℏω)*, and *ℏω* = 24 ± 1 meV, predicting 257 meV at 300 K. Both isolate the same invariants: a large low-*T* width (170 meV) and a polar-mode scale near 25 meV.

The spectral weight *I*_*3*_ *(T)* is almost constant to ∼100 K and then declines slowly. A single-Arrhenius fit yields *E*_*a3*_ = 38 meV, *A* = 2.0, and *I*_*0*_ = 2 × 10^6^ (a.u). The barrier reflects the nearest competing nonradiative pathway within the emitting manifold.

## Time-Resolved Photoluminescence (TRPL)

To connect the steady-state trends to carrier dynamics, we measured TRPL (SI, Figure S7) selecting the three emissive bands in Figure 2. Peak 2 is not resolved at 300 K (Figure 2) and overlaps the high-energy tail of Peak 3, precluding an independent decay.

At 10 K, the decays separate cleanly. Peak 1 is the fastest and multiexponential (spanning 1–800 ns), consistent with radiative recombination from excitonic/weakly localized states^14,15,21–23^ together with nonradiative competition (the rapid quench of *I*_1_(*T*) in Figure 3c). Peak 2 is slower and also multiexponential (spanning 50–1600 ns), indicating a distribution of capture environments and/or feeding from other states,^23,31^ in line with the strong Arrhenius quench (Figure. [Fig fig3]f). Peak 3 is single-exponential and long-lived (3.4 us), matching its weak temperature dependence and modest quench (Figure. [Fig fig3]h).

At 300 K, the hierarchy persists: Peak 1 accelerates as nonradiative pathways strengthen and bound-exciton channels vanish,^14,15,21,22^ whereas Peak 3 remains single-exponential with only a modest reduction, mirroring the gentle decline of *I*_3_(*T)*. Thus, the kinetics reinforce the steady-state picture (Figures 2–3): Peak 1 is governed by temperature-activated losses; Peak 2 is a fragile radiative defect channel that is easily outcompeted at elevated temperature; and Peak 3 arises from a compact, strongly localized emissive defect that remains radiatively efficient even at room temperature. Data and fit results are provided in the SI (Figure S7). The weak temperature and fluence dependence of the Peak-3 lifetime suggests recombination through a compact deep center rather than trap-limited detrapping from a distribution of states.

## Fluence-Dependent Photoluminescence

Figure 4 shows fluence­(*J*)-dependent PL at 10 K. The integrated PL (Figure 4c) is modeled as *I*_PL_ *∝ J* ^*m*^. In doped material *m* = 1 at low injection; deviations reflect trap filling or higher-order channels.^31,33^ **Peak 1** shows *m* ≈ 1.39 at low fluence and *m* ≈ 0.8 at high fluence. At 300 K, where Peak 1 collapses to a single 720 nm band [SI, Figure 9], *m* ≈ 1.55 across the measured range, consistent with trap depopulation as excitation increases.^31^

**Figure 4:**
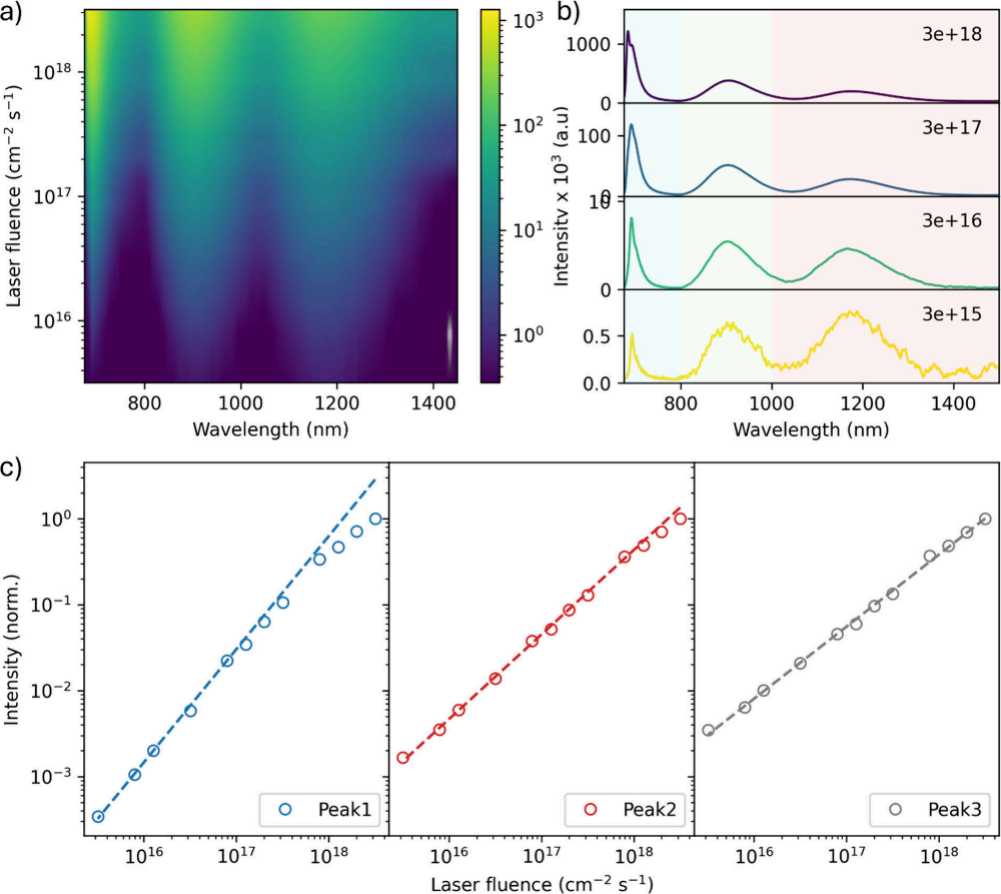
Excitation fluence dependence of PL spectra at 10 K: (a) False-color contour map of PL intensity recorded under above-bandgap 532 nm excitation, (b) Representative spectra extracted from panel (a) at four different fluences (photons/cm^2^/s), (c) Integrated PL intensity as a function of excitation fluence for the three peaks, along with power-law fit *I*_PL_ ∝ *J* ^*m*^. Note that Peak 1 resolves into several narrow components (SI, Figure S8), as discussed previously in the context of Figure 2; with increasing fluence, the 681 nm (near-edge) component grows relative to the 690 and 703 nm lines.

For **Peak 2**, the center and fwhm are fluence-independent within resolution (SI, Figure S10), arguing against carrier-density–induced renormalization. The weight rolls from m ≈ 0.98 at low fluence to m ≈ 0.76 at high fluence (Figure 4c), consistent with a finite active-center population.^33^ At 300 K the peak is not discernible (Figure 2b), in line with the 0.16 eV Arrhenius quench (Figure. [Fig fig3]f).

**Peak 3** remains broad; its center and width vary weakly with fluence. At 10 K, the center red-shifts by ∼8 meV and the fwhm grows by ∼19 meV. In contrast, at 300 K, the center blue-shifts by ∼13 meV with ∼12 meV broadening. The weight scales sublinearly over the full range (*m* ≈ 0.84 at 10 K, *m* ≈ 0.82 at 300 K). The small shifts are captured (SI, Figure S11) by^33,35^

7 $$E_{3}\left(J\right)=E_{0}-\frac{\Delta }{1+J/J_{0}}$$

8 $$FWHM\left(J\right)={FWHM}_{0}+\Delta \left[1-e^{-J/J_{0}}\right]$$

with E_0_ = 1.055 eV, Δ = −8 meV, *J*_*0*_ = 1.5 × 10^17^ cm^–2^/s (center, 10 K) and fwhm_0_ = 149 meV, Δ = 19 meV, and J0 = 4 × 10^17^ cm^–2^/s (width). The similar *J*_*0*_ scales point to a common density threshold for shift and broadening. At 300 K, the same equations with Δ > 0 describe the modest blue shift (SI, Figure S12).

## Hyperspectral Photoluminescence Mapping

To link ensemble trends to microstructure, we performed hyperspectral confocal PL mapping.^36,37^ The band-edge–filtered intensity map (Figure 5a) resolves the polycrystalline texture: emission is strongest on grains and suppressed along boundaries, with measurable variation within single grains. The peak-center map (Figure 5b) shows a frequent blue-shift near edge-rich regions (small grains/boundaries) relative to the 720 nm average, consistent with microstructure-correlated local heterogeneity (e.g., electrostatic potential fluctuations and/or disorder-related band-tail effects) near edge-rich regions; strain- or carrier-density–driven contributions are not independently resolved in the present data.^20,31,33,34^ The band-edge line width (Figure 5c) is broader in boundaries/intergranular regions and narrower in large-grain interiors, mirroring the inhomogeneous broadening inferred from the temperature-dependent analysis (Figure 3): static disorder and local field fluctuations dominate where the microstructure is edge-rich.^31,35^

**Figure 5:**
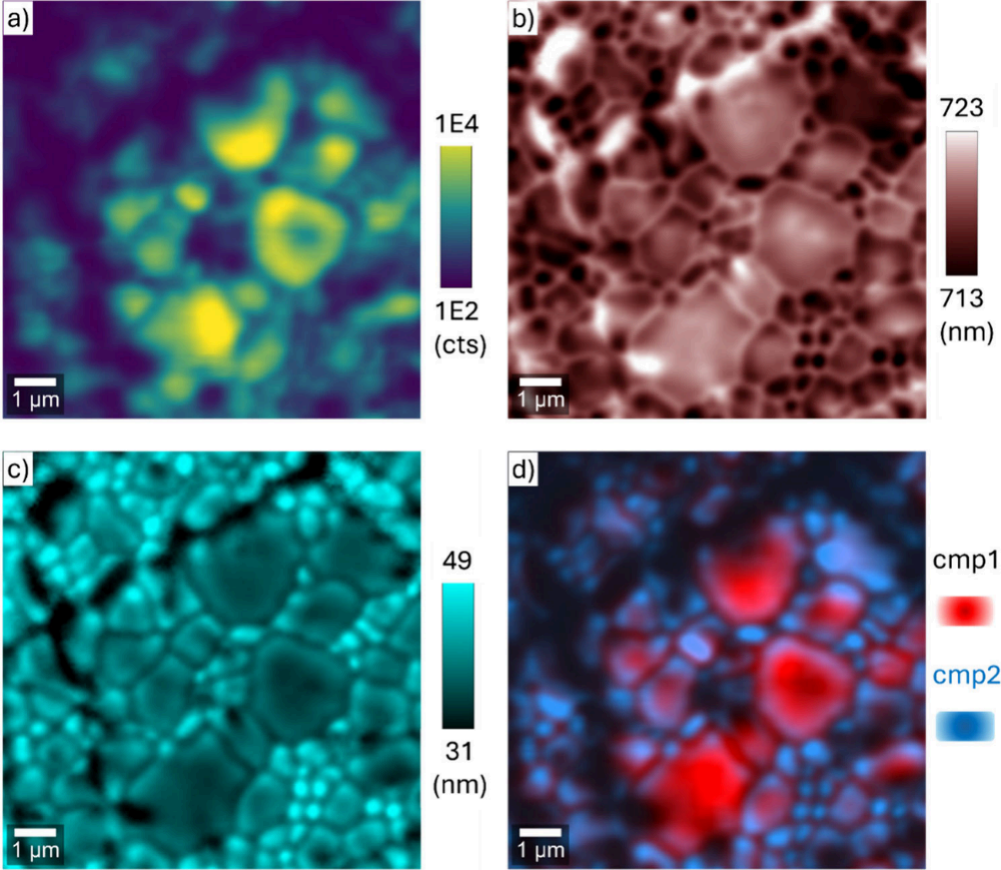
Hyperspectral micro-PL maps measured at 300 K, filtered to band-edge Peak 1: spatial variation of (a) peak intensity, (b) peak center wavelength, (c) *FWHM*, and (d) overlaid spectral components (the component spectra are given in the SI, Figure S13).

To assess spectral heterogeneity, we fitted the full map using two component spectra extracted from the data set (SI, Figure S13). The reconstructed map (Figure 5d) shows that the component with a stronger infrared tail (>850 nm) is enriched at grain boundaries, whereas the component with comparatively stronger near-edge emission dominates grain interiors (SI, Figure S13). Because the detector response rolls off beyond ≈1030 nm, we do not claim full spatial localization of the 1.05 eV band; rather, within the instrument window the relative long-wavelength weight is higher in edge-rich regions. Together, these maps show that the infrared defect emission is dominant at edge-rich regions, where the band-edge PL is attenuated, blue-shifted, and broadened.

## First-Principles Calculations

First-principles density functional theory (DFT) calculations were used to determine formation energies and transition levels of intrinsic defects and Cl impurities in CdSe (Figure 6a–b, SI, Figure 16). Motivated by CdCl_2_ activation, we focused on Cl substitution at the Se site (Cl_Se_), the lowest-energy configuration.^38^ Cl_Se_ exhibits very low formation energies (≤1.5 eV Se-rich; ≤0.5 eV Cd-rich), with a +/0 level resonant in the conduction band. It donates an electron upon formation, emerging as the dominant n-type dopant that pins the Fermi level near the CBM and dictates the stability of native defects.

**Figure 6:**
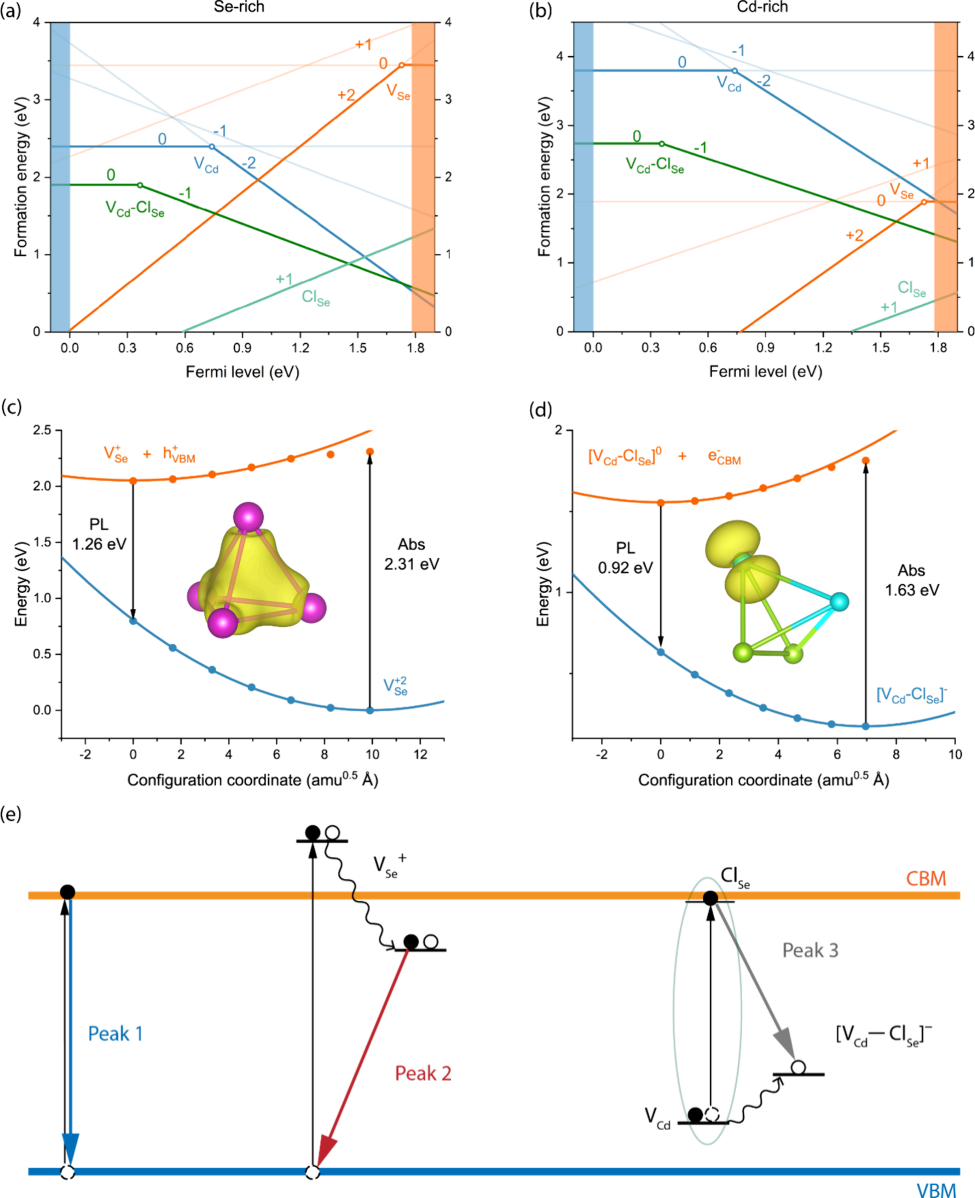
First-principles DFT calculations of point defects in CdSe. Defect formation energies under (a) Se-rich and (b) Cd-rich conditions, where partially transparent lines denote thermodynamically unstable charge states of *V_Se_* and *V*_*Cd*_. The blue and orange areas denote valence bands and conduction bands, respectively. Configuration coordinate diagrams for (c) *V*_*Se*_^+^ capturing a hole from the valence-band maximum (VBM) and (d) [*V*_*Cd*_ – *Cl*_*Se*_]^0^ capturing an electron from the conduction-band minimum (CBM), illustrating the radiative recombination pathways. Solid circles represent calculated data points, and curves are parabolic fits. Insets show the atomic structures of (c) *V*_*Se*_^+^ and (d) [*V*_*Cd*_ – *Cl*_*Se*_]^0^ with ball-and-stick representations and charge density isosurfaces. Pink, green, and cyan spheres denote Cd, Se, and Cl atoms, respectively. (e) Band diagram illustrating the excitation and emission processes corresponding to the three peaks observed in the experiment. Up/down arrow denotes excitation/emission process. Curly lines represent lattice relaxation. Solid/empty circle denotes electron/hole. Dashed circles indicate holes left behind due to photoexcitation.

Among intrinsic defects, cation and anion vacancies dominate. Both V_Cd_ and V_Se_ show negative-U behavior. Under n-type conditions, V_Cd_ forms readily (<1 eV Se-rich, ∼2 eV Cd-rich) with a deep 0/–2 level at E_V_+0.74 eV, deeper than in CdTe and indicative of strong nonradiative recombination.^39^ V_Se_ has comparable energy under Cd-rich but is suppressed in Se-rich; its +2/0 level lies only ∼60 meV below the CBM, acting as a shallow double donor that further supports n-type conductivity.

Configuration-coordinate diagrams rationalize the observed sub-gap PL (Figure 6c–d). Peak 2 originates from V_Se_^+^: after above-gap excitation, an electron is trapped in the localized +1 state (Inset) and radiatively recombines with a hole, yielding 1.26 eV emission—consistent with experiment and absent under 785 nm pumping. Peak 3 is consistent with a compact [V_Cd_–Cl_Se_ ] donor–acceptor complex. When Cl substitutes a Se neighbor of V_Cd_ (Inset), charge transfer creates a bound pair with lower formation energy than isolated V_Cd_. This complex introduces a 0/– level at E_V_+0.36 eV, and produces ∼0.92 eV emission with strong vibronic coupling, matching the large line width and vibrational scale inferred experimentally (Figure 3h).

Together, these results support the experimental assignment (Figure 6e): band-edge emission (Peak 1), V_Se_-mediated above-gap-fed luminescence (Peak 2), and a robust, vibronically active [V_Cd_–Cl_Se_ ] complex underlying Peak 3. Oxygen-related defects or complexes were not included in the present DFT calculations; since CdCl_2_ activation is not oxygen-free, oxygen-mediated variants of the deep emissive center cannot be excluded.

CdCl_2_ activation couples microstructure to optics in CdSe: it converts porous nanograins into dense micrometer-scale grains, sharpens the band edge (Urbach 85→17 meV at 300 K), and exposes three emissive channels with distinct kinetics and temperature responses. The near-edge band (Peak 1) evolves from excitonic to free-carrier emission and is governed by a ∼25 meV polar phonon; a band at *E*_*g*_ −0.45 eV (Peak 2) tracks *E*_*g*_ *(T)* and quenches with ∼0.16 eV; and a ∼1.05 eV band (Peak 3), excited by above- and below-gap photons, blue-shifts with temperature and sustains microsecond lifetimes at room temperature. Hyperspectral maps link enhanced infrared emission to edge-rich regions where band-edge PL is suppressed and broadened. Hybrid-DFT supports chlorine donors, a selenium-vacancy route for the sub-gap band, and a plausible cadmium-vacancy–chlorine complex for the infrared band.^41^ Future work combining PL with advanced contactless spectroscopies^40^ and systematically varying CdCl_2_ treatment conditions, ideally in conjunction with device performance metrics, will be valuable for further constraining the microscopic origin and impact of chlorine-related deep infrared emission.

## Supplementary Material



## Notes

Experimental methods, first-principles calculation details, supplementary figures and tables, Urbach energy extraction, absorption spectra, and supplementary references. (PDF)

## References

[1] Shelke N. T., Karle S. C., Karche B. R. (2020). Photoresponse properties of CdSe
thin film photodetector. J. Mater. Sci.: Mater.
Electron..

[2] Jang E., Jang H. (2023). Review: Quantum
Dot Light-Emitting Diodes. Chem. Rev..

[3] Salem M. S. (2024). Design considerations
of CdSe solar cells for indoor applications. Sol. Energy Mater. Sol. Cells.

[4] Bastola, E. ; Cadmium Selenide (CdSe) as an Active Absorber Layer for Solar Cells with Voc Approaching 750 mV. Proc. 2023 IEEE PVSC (2023). 10.1109/PVSC48320.2023.10359777

[5] Hill, T. ; Grover, S. ; Sites, J. Widegap CdSe Solar Cells with VOC >750 mV. Proc. 2023 IEEE PVSC (2023). 10.1109/PVSC48320.2023.10359709

[6] Li K., Lin X., Song B. (2021). Rapid thermal evaporation for cadmium selenide
thin-film solar cells. Front. Optoelectron..

[7] Li K., Yang X., Lu Y., Xue J., Lu S., Zheng J., Chen C., Tang J. (2022). Fabrication
and Optimization
of CdSe Solar Cells for Possible Top Cell of Silicon-Based Tandem
Devices. Adv. Energy Mater..

[8] Poplawsky J. D. (2016). Structural and compositional dependence of the CdTex Se1–x
alloy layer photoactivity in CdTe-based solar cells. Nat. Commun..

[9] Li D.-B. (2022). 20%-efficient polycrystalline
Cd­(Se,Te) thin-film solar cells with
compositional gradient near the front junction. Nat. Commun..

[10] Liu X. (2024). The effect of remnant
CdSe layers on the performance of CdSeTe/CdTe
photovoltaic devices. Sol. Energy Mater. Sol.
Cells.

[11] Fang H., Hu W. (2017). Photogating in Low-Dimensional
Photodetectors. Adv. Sci..

[12] Bowman A. R. (2024). ″Spatially resolved photoluminescence analysis of the role
of Se in CdSexTe1– x thin films.″. Nat. Commun..

[13] Altamimi T. F. S. (2024). ″Se Inter-Diffusion
Limits Absorber Layer Grain
Growth in Cd Se-Cd Te Photovoltaics.″. PRX Energy.

[14] Wojtowicz-Natanson B., Zakrzewski T. (1965). Temperature
Dependence of the Spectrum of the Photoluminescence
in CdSe Crystals. phys. stat. sol..

[15] Gross E. F., Razbirin B. S., Fedorov V. P., Naumov, Yu P. (1968). Bound Exciton
Complexes
in CdSe Crystals. phys. stat. sol..

[16] Rosen D. L., Li Q. X., Alfano R. R. (1985). Native
defects in undoped semi-insulating
CdSe studied by photoluminescence and absorption. Phys. Rev. B.

[17] Brasil M. J. S. P., Motisuke P., Decker F., Morrot J. R. (1988). Infrared photoluminescence
at deep centres in polycrystalline CdSe layers. J. Phys. C: Solid State Phys..

[18] Gracia-Jiménez J. M., Martínez-Montes G., Silva-González R. (1992). Photoluminescence
of Chemical Bath Deposited CdSe Films. J. Electrochem.
Soc..

[19] Mahmoud S., Eid A. H. (1990). Microstructure optical
and electrical studies of cadmium
selenide thin films. Cryst. Res. Technol..

[20] Ndiaye A., Youma I. (2003). Effects of post-deposition
processing on microstructural properties
of CdSe thin films grown from solution. Eur.
Phys. J. Appl. Phys..

[21] Gourdon C., Lavallard P., Dagenais M. (1988). Time dynamics of free- and bound-exciton
luminescence in CdSe under low- and high-intensity excitation. Phys. Rev. B.

[22] Minami F., Era K. (1985). Lifetimes of bound excitons in CdSe. Solid
State Commun..

[23] Shumaker M. L., Dollard W. J., Waldeck D. H. (1992). Carrier relaxation at semiconductor
interfaces and essential features of a quantitative model (TRPL of
CdSe). J. Phys. Chem..

[24] Kuciauskas D. (2022). ″Voltage loss
comparison in CdSe/CdTe solar cells and polycrystalline
CdSeTe heterostructures.″. IEEE Journal
of Photovoltaics.

[25] Abbassi A. (2015). “Comparative study of cubic and wurtzite CdSe.”. SpringerPlus.

[26] Varshni Y. P. (1967). Temperature
dependence of the energy gap in semiconductors. Physica.

[27] Adachi, S. Properties of Group-IV, III-V and II-VI Semiconductors. (Wiley, 2005).

[28] Pässler R. (2003). Semi-empirical
descriptions of temperature dependences of band gaps in semiconductors. phys. stat. sol. (b).

[29] Rudin S., Reinecke T. L., Segall B. (1990). Temperature-dependent exciton linewidths
in semiconductors due to LO- and acoustic-phonon scattering. Phys. Rev. B.

[30] Milekhin I. A., Milekhin A. G., Zahn D. R. T. (2022). Surface- and Tip-Enhanced
Raman Scattering by CdSe Nanocrystals: LO phonon near ∼ 210
cm^–1^ (≈ 26 meV) dominates bulk CdSe. Nanomaterials.

[31] Klingshirn, C. Semiconductor Optics, 4th ed. (Springer, 2012).

[32] O’Donnell K. P., Chen X. (1991). Temperature dependence of semiconductor band gaps. Appl. Phys. Lett..

[33] Pankove, J. I. Optical Processes in Semiconductors. (Prentice-Hall, 1971).

[34] Henderson, B. ; Imbusch, G. F. Optical Spectroscopy of Inorganic Solids. (Clarendon/OUP, 1989).

[35] Reshchikov M. A., Morkoç H. (2005). Luminescence
properties of defects in GaN. J. Appl. Phys..

[36] Abudulimu A., Liu L., Liu G., Aimaiti N., Rezek B., Chen Q. (2020). Crucial role
of charge transporting layers on ion migration in perovskite solar
cells. Journal of Energy chemistry.

[37] Abudulimu, A. ; ″Photophysical properties of CdSe/CdTe bilayer solar cells: a confocal raman and photoluminescence microscopy study.″ 2022 IEEE 49th Photovoltaics Specialists Conference (PVSC). IEEE, (2022).

[38] Mannodi-Kanakkithodi A., Toriyama M. Y., Sen F. G. (2020). Machine-learned
impurity
level prediction for semiconductors: the example of Cd-based chalcogenides. npj Comput. Mater..

[39] Kavanagh S. R., Walsh A., Scanlon D. O. (2021). Rapid Recombination by Cadmium Vacancies
in CdTe. ACS Energy Letters.

[41] Kuciauskas D., Hill T., Sites J. R., Grover S., Tong Y., Dunham S. T. (2025). Increased Voltage
in CdSe Solar Cells by Mitigation
of Charge Carrier Trapping Due to Se Vacancies. Adv. Mater. Technol..

[40] Kuciauskas D. (2025). ″Doping with
phosphorus reduces anion vacancy disorder in
CdSeTe semiconductors enabling higher solar cell efficiency.″. Nat. Commun..

