# Supplementary material for: Radiative Defects in Chloride-Activated CdSe Thin Films
Source: ACS Energy Lett. 2026 Jan 26;11(2):1696–704. doi: 10.1021/acsenergylett.5c03191 (PMC12910657; doi:10.1021/acsenergylett.5c03191)
Supplement: Supplementary file 1 [file nz5c03191_si_001.pdf]

Supporting Information

Radiative defects in chloride-activated CdSe thin films

Abasi Abudulimu[1], Xiaoming Wang[1], Tyler Brau[1], Jaroslav Kuliček[2], Scott L. Wenner[1], Adam B. Phillips[1], Ebin Bastola[1], Manoj K. Jamarkattel[1], Vijay Karade[1], Kiran Lamichhane[1], Aparajita Dixit[1], Bohuslav Rezek[2], Yanfa Yan[1], Michael J. Heben[1], Randy J. Ellingson[1*]*

[1]Wright Center for Photovoltaics Innovation and Commercialization (PVIC), Department of Physics and Astronomy, The University of Toledo, OH 43606 USA

[2]Faculty of Electrical Engineering, Czech Technical University in Prague,
Technická 2, 16627 Prague, Czechia

E-mail: abasi.abudulimu@utoledo.edu
randy.ellingson@utoledo.edu

Table of contents

1. Experimental Methods: page 2
2. DFT Calculation Details: page 3
3. Supplementary Figures: pages 4-17
4. Supplementary Table: page 18
5. Urbach Energy Extraction: page 19
6. Absorption Spectra: page 19
7. Supplementary References 1-19: page 20

1. Experimental section

Cadmium selenide (CdSe) films were deposited on a TEC12D glass substrate received from Pilkington (NSG Group). The glasses were cleaned using a Micro-90 detergent under ultrasonic cleaning and a few rinses with deionized water. Source material for the CdSe was received from 5N plus. CdSe films was deposited using a thermal evaporation technique at a base pressure of 10^{-6} Torr at a substrate temperature of 400 ℃. CdSe film was cadmium chloride ($CdCl_2$) treated with saturated $CdCl_2$ solution in methanol as reported previously [1]. For a sample of 3”x3”, $CdCl_2$ solution of 0.6 mL was drop-casted, and let it dry in the fume hood. Then, the film was heated at 450 ℃ for 40 mins in a dry-air environment. CdSe film was rinsed with methanol, dried with dry-air followed by other characterizations. The optical absorption spectra of CdSe films were determined from measured reflectance and transmittance using a PerkinElmer Lambda 1050 spectrophotometer.

The surfaces and cross-sectional images of the CdSe films were captured using a Hitachi S-4800 UHR scanning electron microscope (SEM). X-ray diffraction patterns were obtained using a Rigaku Ultima III X-ray diffractometer equipped with a Cu k-alpha source operating at a 40 kV accelerating voltage and a current of 44 mA.

Photoluminescence (PL) and time-resolved PL (TRPL) were measured with the sample mounted in a closed-cycle helium cryostat (Advanced Research Systems DE-202-AI) equipped with a LakeShore 335 temperature controller. The cryostat chamber was evacuated for 30 min using a Pfeiffer TSH-071-E pump before cooling with an ARS-2HW helium compressor. Continuous-wave PL excitation was provided by a Spectra-Physics Excelsior 532 nm laser and a Thorlabs 785 nm laser diode. Pulsed TRPL excitation was provided by a Fianium supercontinuum source, with 532 nm or 785 nm wavelengths selected by an acousto-optic tunable filter. Long-pass filters (665 nm for 532 nm excitation and 850 nm for 785 nm excitation) were used to block the laser excitations reaching the detector. Spectra were collected with LN_2-cooled detectors, a Si CCD and a Ge array, and corrected for system response using a calibrated tungsten–halogen lamp. Unless otherwise specified, the excitation fluence was fixed at 5 × 10^17 photons cm^{-2} s^{-1} for temperature-dependent PL and TRPL. Injection-dependent PL was obtained by varying the fluence between 5 × 10^15 and 5 × 10^18 photons cm^{-2} s^{-1} using neutral-density filters. Further details of the PL/TRPL system are described elsewhere [2-3].

Hyperspectral micro-photoluminescence (PL) maps were recorded using a confocal Raman/PL microscope (WITec alpha300 RAS) equipped with a quasi-tunable white light laser source (NTK Photonics) with excitation wavelength 520 nm and laser power 130 µW [4-5]. A long-pass filter with a cutoff of 550 nm was placed before the detector to filter the signal from the laser. Spectral data were captured through a 100x objective lens (NA/0.9) and analyzed using a UHTS 300 VIS spectrometer. An X-Y piezo flexure stage was used for the sample lateral scanning for obtaining the PL spectral maps in the areas: 2.5 × 2.5 µm2, scan speed 26 s/line, 512 × 512 pixels per image. PL maps were recorded and processed by WITec Control 6.1 and WITec Project 6.1 software.

2. DFT calculation details

All first-principles calculations were performed using the VASP code [6-8] with the projector-augmented wave (PAW) method [9]. The zinc-blende phase of CdSe, containing two atoms per unit cell, was adopted as the reference structure. A plane-wave cutoff energy of 300 eV and a Γ-centered 4×4×4 Monkhorst–Pack k-point mesh were used for total-energy and electronic-structure calculations. The Heyd–Scuseria–Ernzerhof (HSE06) hybrid functional [10-11] with 32% exact exchange was employed to reproduce the experimental band gap, while the PBE functional [12] with a denser Γ-centered 16×16×16 mesh was used for dielectric constant calculations. Structural optimizations of both bulk and defect supercells were performed until the residual forces on all atoms were less than 0.01 eV/Å.

Defect calculations were carried out in a 64-atom supercell, constructed from a 2×2×2 expansion of the conventional cubic CdSe unit cell. A Γ-centered 2×2×2 k-point mesh was used for Brillouin-zone sampling. Finite-size effects in charged defect calculations were corrected using the extended Freysoldt–Neugebauer–Van de Walle (FNV) scheme [13-14]. Optical transition energies were further corrected with the GKFO scheme [15], while Lany–Zunger 2/3 scaling of the point-charge correction [16] was applied to all charge corrections. The *doped* [17] and *pydefect* [18] packages were employed to assist defect energetics and analysis.

3. Supplementary Figures

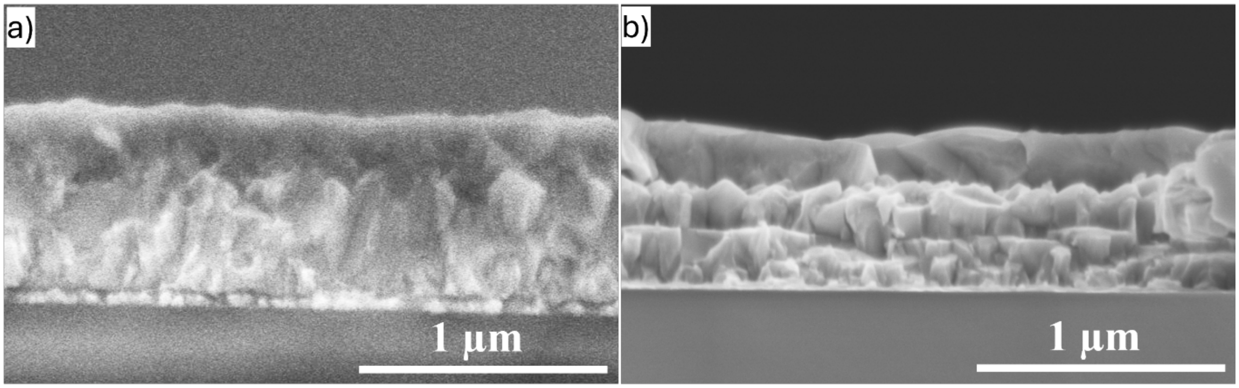

Supplementary Fig. 1. Cross-sectional SEM images of (a) as-deposited and (b) CdCl2 treated CdSe thin film. Estimated film thickness is about 300 nm.

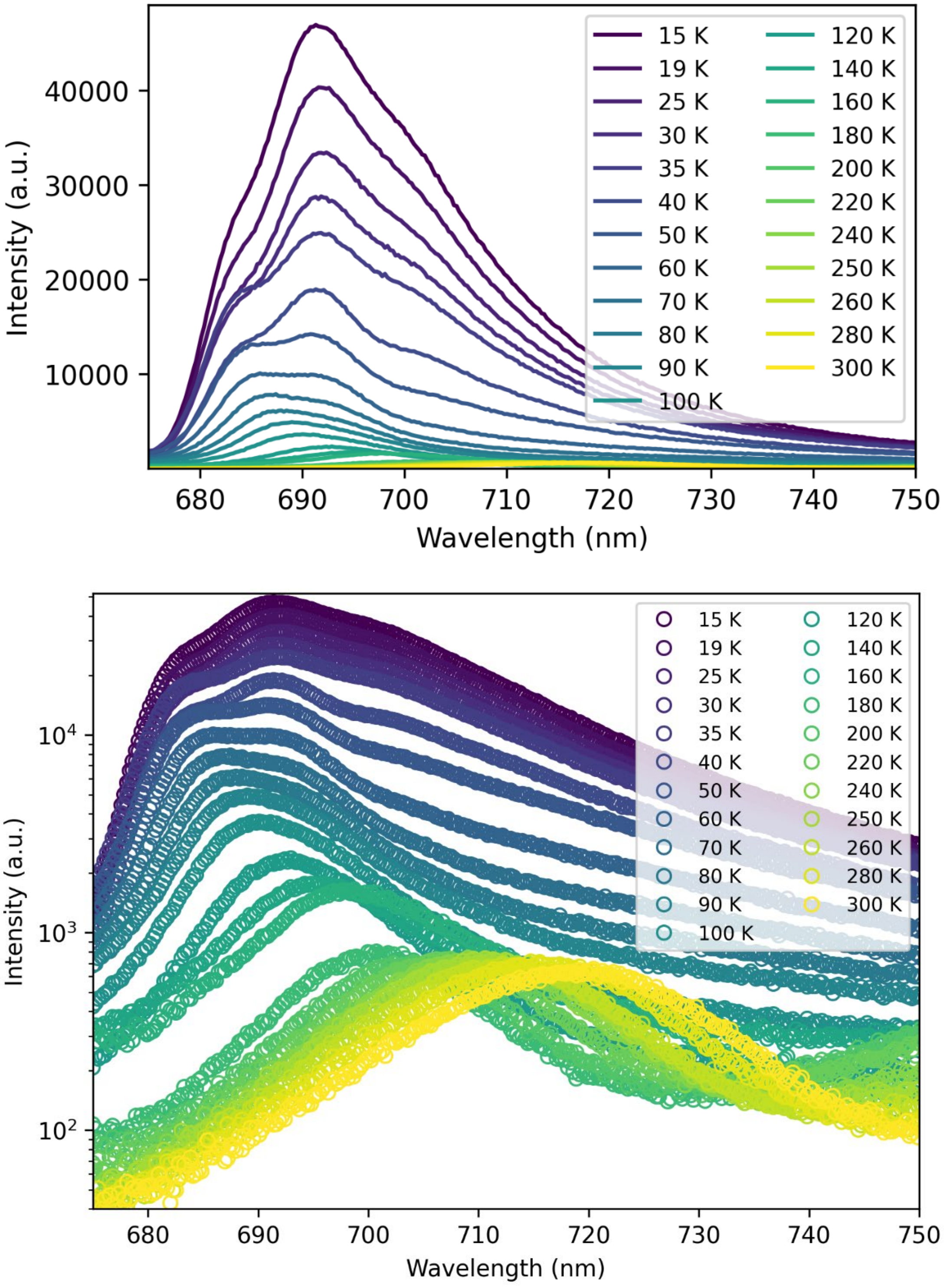

Supplementary Fig. 2. Temperature-dependence of near-edge band (Peak 1) measured from 15 K--300 K under 532 nm laser excitation, given in linear-linear and linear-log scale. Three peaks (681, 690, 703 nm) present below 80 K, taken over by 681 nm above 80 K and the peak center red-shifts 720 nm by 300 K.

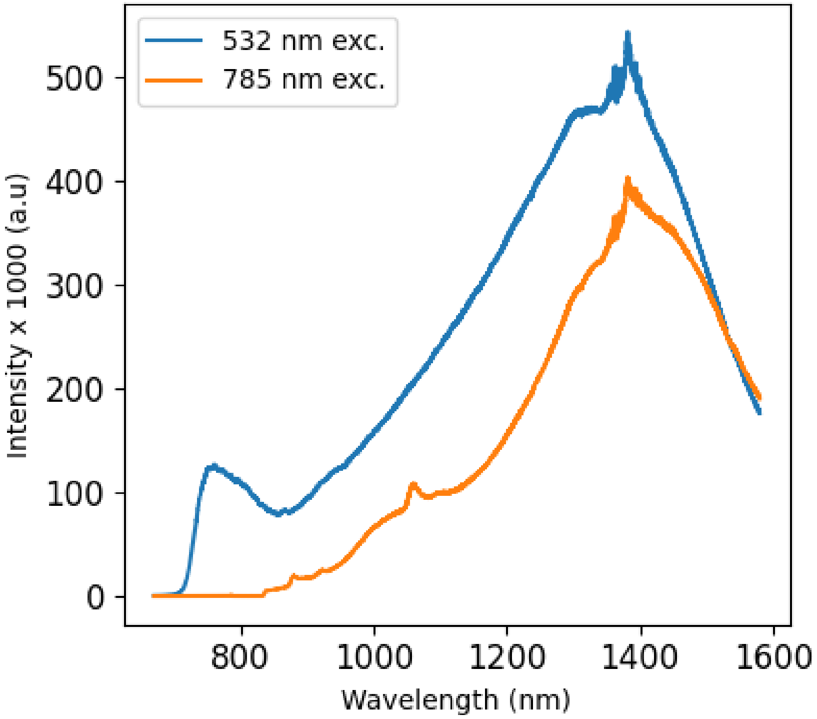

Supplementary Fig. 3. PL spectra of as-deposited CdSe film measured at 10 K under 532 nm and 785 nm excitations (fluence: $5x10^{18}$ photons/cm^2/s). The peak around 775 nm is the near-edge band. The peak-like noise between 1370 – 1390 nm is from the noise in the spectral correction. No PL signal was detected at 300 K.

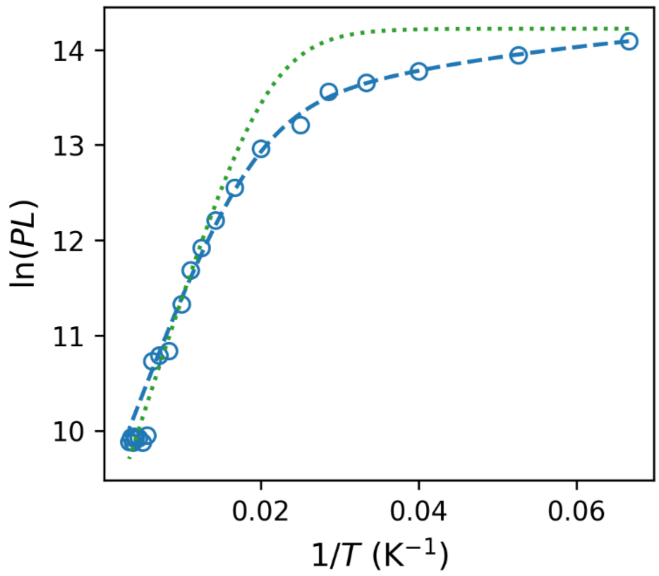

Supplementary Fig. 4. Integrated spectral weight of Peak 1 (symbols) fitted with a single Arrhenius model (green dotted line) and a double Arrhenius model (blue dashed line). The single Arrhenius fit is applied for T> 120 K, while the double Arrhenius fit covers the entire temperature range of 15–300 K. The double-Arrhenius fit yields activation energies E_a = 19.3 meV and E_b = 2.6 meV; the single-Arrhenius fit yields E_a = 22.1 meV.

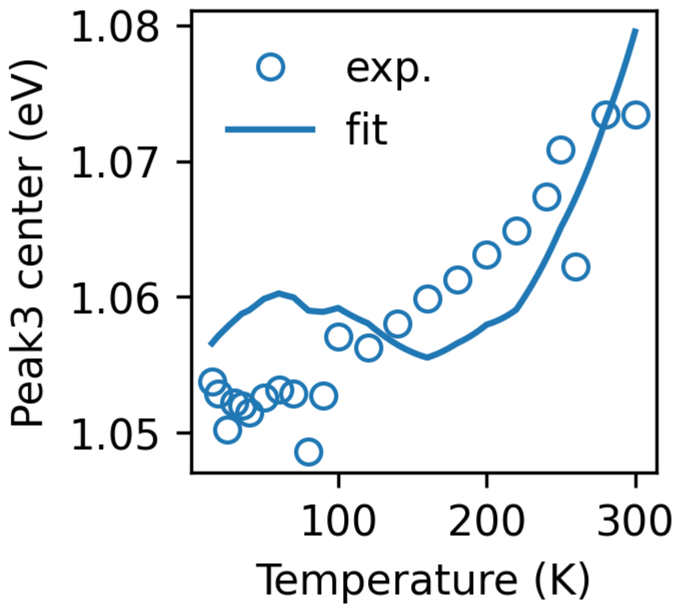

Supplementary Fig. 5 Temperature dependence of the Peak 3 center energy, fitted using conventional donor–acceptor pair expressions that account for the bandgap energy, donor energy, acceptor energy, and a Coulomb term [19].

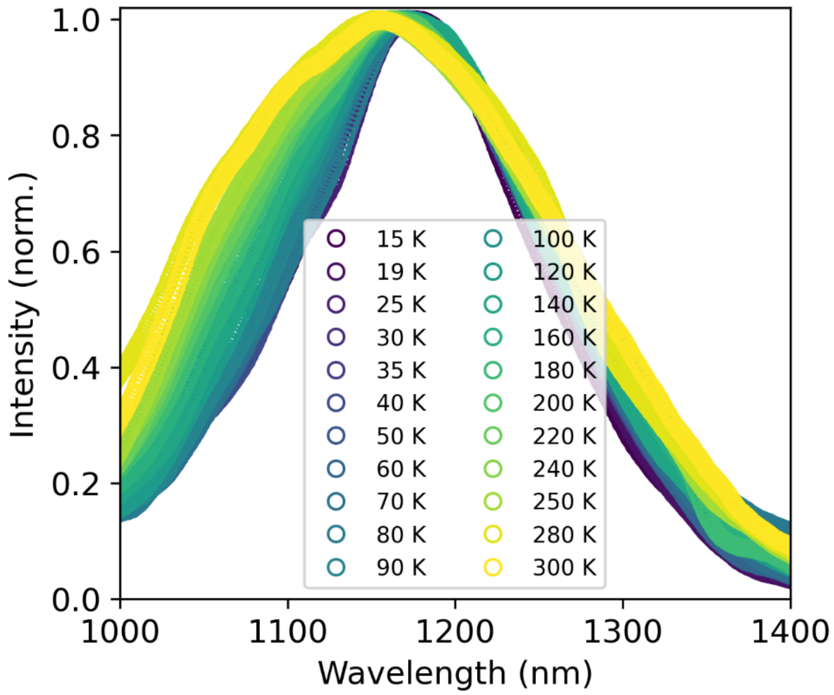

Supplementary Fig. 6. Peak 3 broadening with temperature: higher energy (shorter wavelength) side contributes more to the broadening.

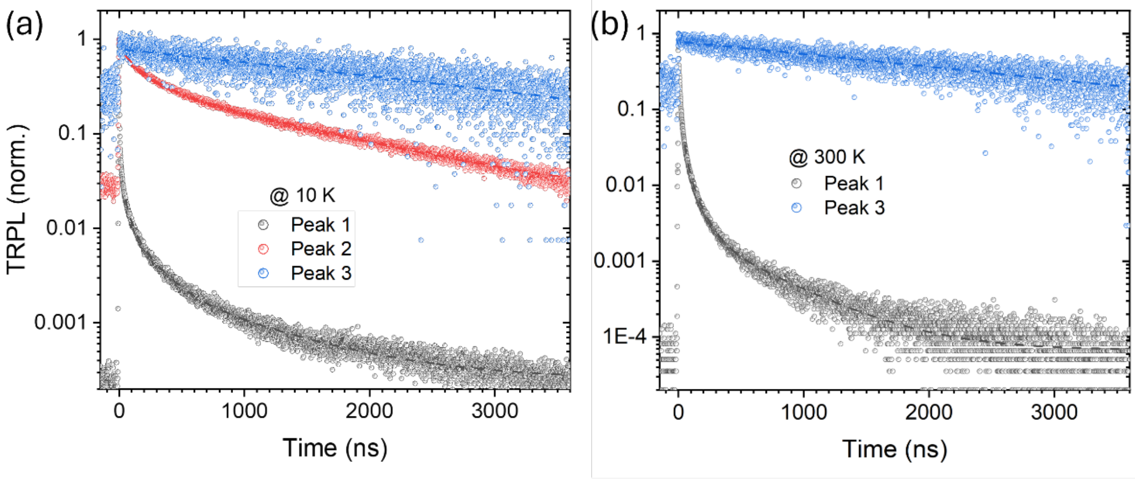

(c)

T = 10 K	*τ_1 (ns)*	*A1 (a.u)*	*τ_2 (ns)*	*A2 (a.u)*	*τ_3 (ns)*	*A3 (a.u)*
Peak 1	42	0.52	173	0.38	806	0.1
Peak 2	52	0.32	280	0.39	1645	0.29
Peak 3	-	1.0	-	-	3417	-
T = 300 K						
Peak 1	26	0.63	89	0.32	503	0.05
Peak 2	-	-	-	-	-	-
Peak 3	-	1.0	-	-	2684	-

Supplementary Fig. 7 Normalized TRPL decay traces measured at (a) 10 K and (b) 300 K under 532 nm laser excitation, and (c) the extracted lifetimes (and the corresponding coefficients: A1, A2, and A3) obtained from single-exponential fits (Peak 3) and multi-exponential fits (Peaks 2 and 3).

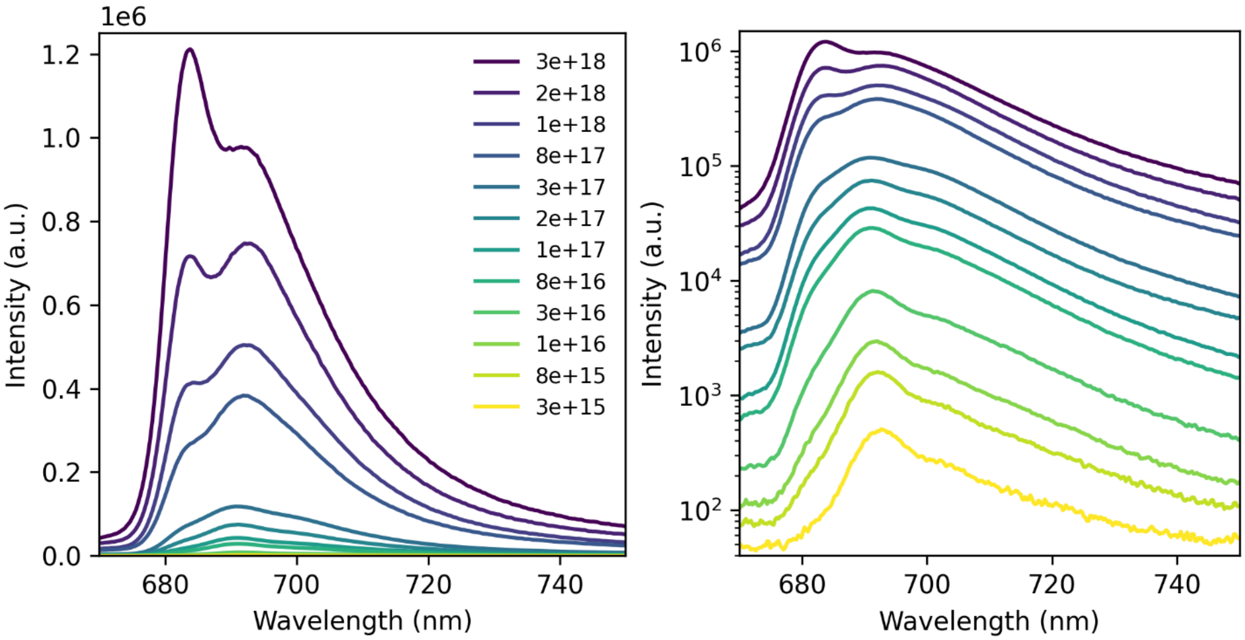

*Supplementary Fig. 8. Excitation fluence dependence of peak 1 at 10 K. Fluence is given in units of photons cm^{-2} s^{-1}. Note that Peak 1 resolves into several narrow components (**SI, Fig. S8**), same as Supplementary Fig. 2; with increasing fluence, the 681 nm (near-edge) component grows relative to the 690 nm and 703 nm lines.*

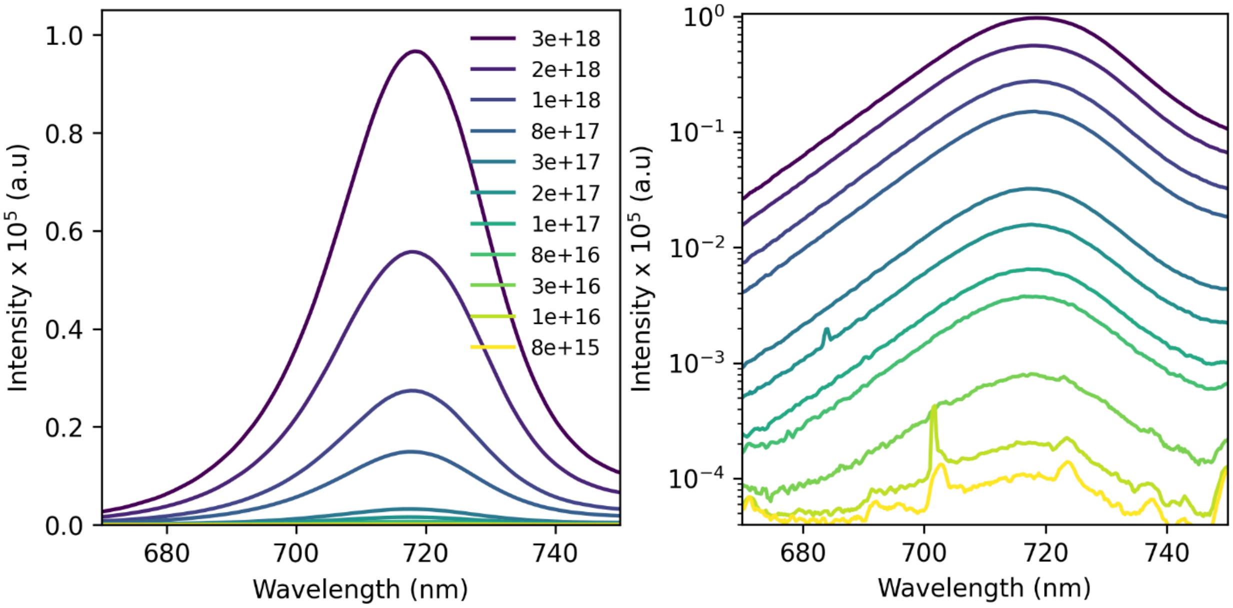

Supplementary Fig. 9. Excitation fluence dependence of peak 1 at 300 K. Fluence is given in units of photons cm^{-2} s^{-1}.

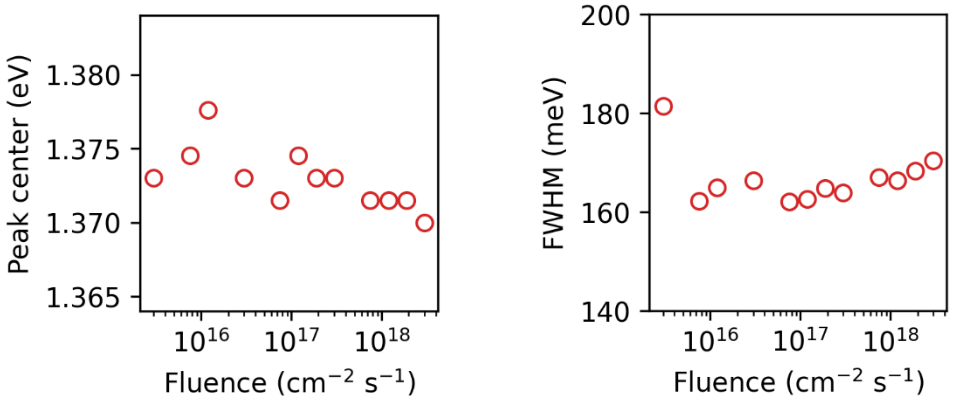

Supplementary Fig. 10. Excitation fluence dependence of peak 2 center energy (left panel) and width (right panel) at 10 K.

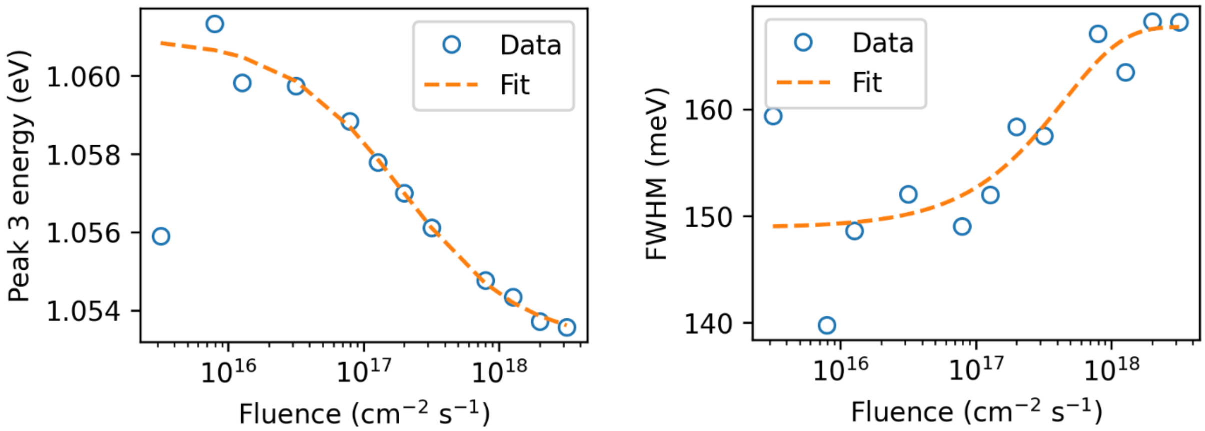

Supplementary Fig. 11. Excitation fluence dependence of peak 3 center energy (left panel) and width (right panel) at 10 K.

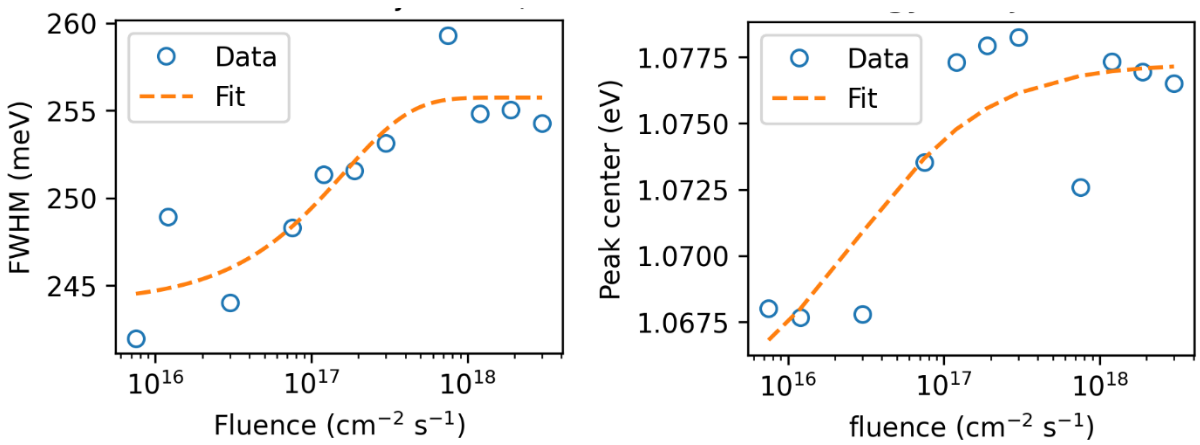

Supplementary Fig. 12. Excitation fluence dependence of peak 3 center energy (left panel) and width (right panel) at 300 K.

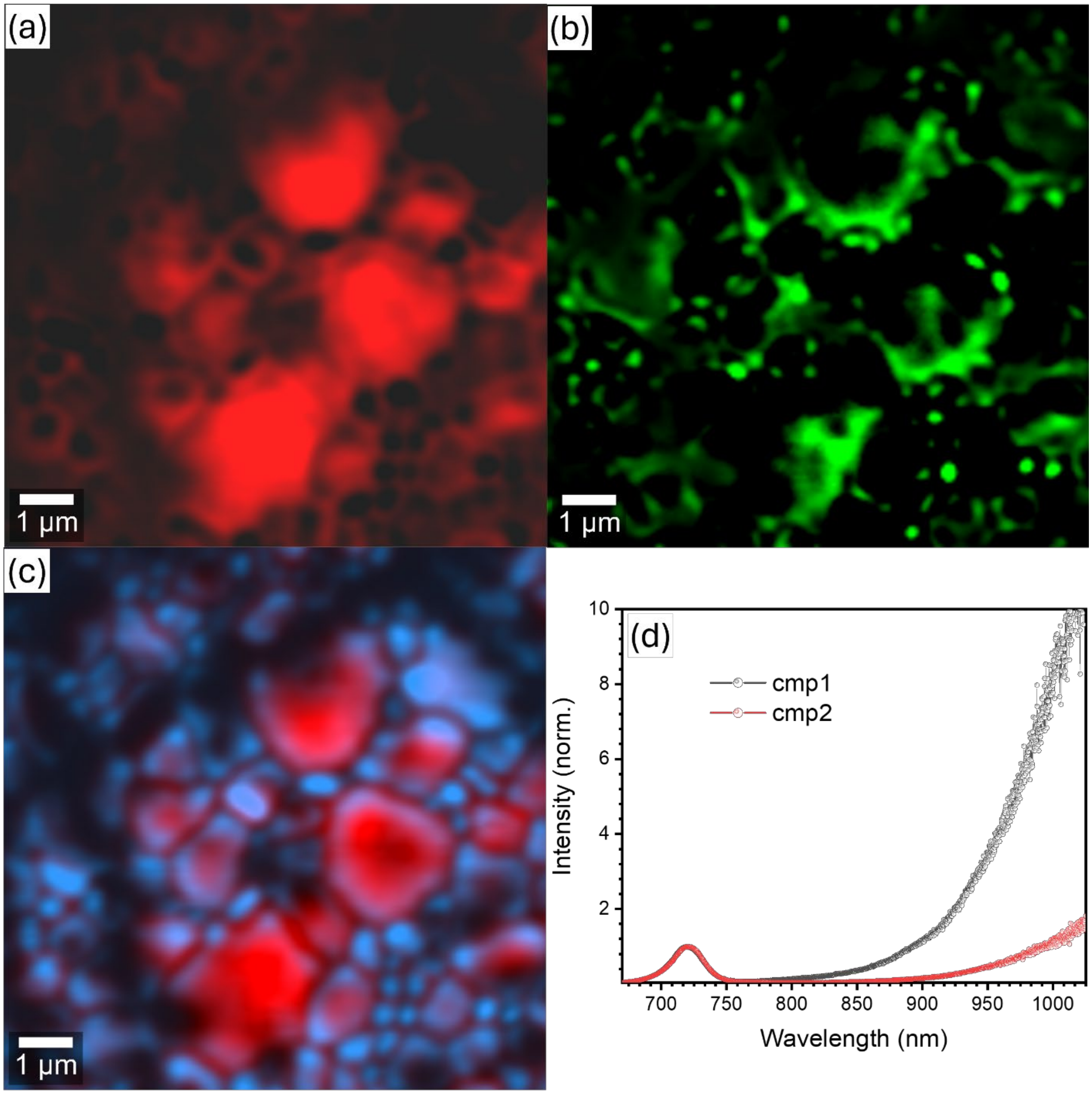

Supplementary Fig. 13. Principal component analysis of the hyperspectral PL map using two component spectra (d). The resulting distribution maps correspond to component 1 (a) and component 2 (b), with (c) showing the overlaid map of components 1 and 2.

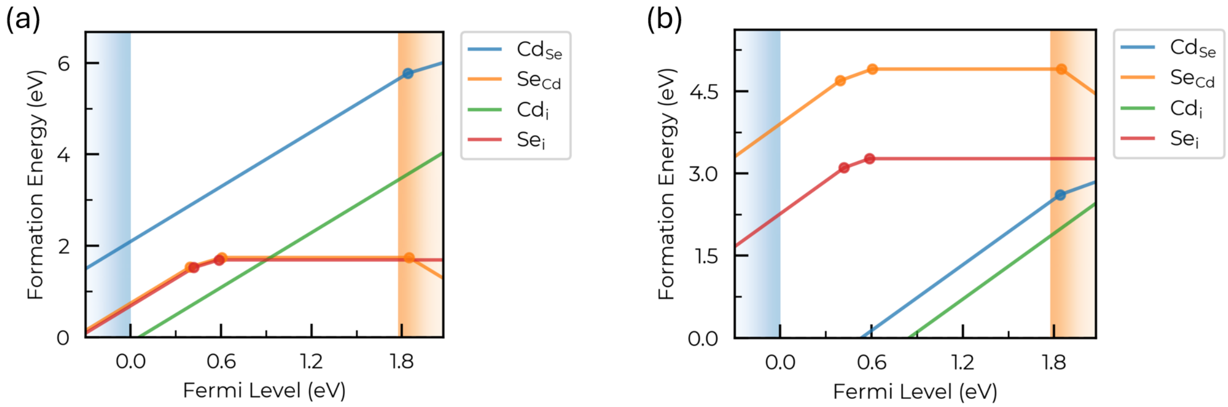

Supplementary Fig. 14. Defect formation energies of CdSe under (a) Se-rich and (b) Cd-rich conditions.

4. Supplementary Tables

Table I: Sub-band gap transitions and emission energies of point defects in CdSe.

Transition	E_{PL} (eV)
$\mathrm{V_{Se}^{+}} + h_{\mathrm{VBM}}^{+} \rightarrow \mathrm{V_{Se}^{+2}}$	1.26
$[\mathrm{V_{Cd}} - \mathrm{Cl_{Se}}]^{0} + e_{\mathrm{CBM}}^{-} \rightarrow [\mathrm{V_{Cd}} - \mathrm{Cl_{Se}}]^{-}$	0.92
$\mathrm{V_{Cd}^{-}} + e_{\mathrm{CBM}}^{-} \rightarrow \mathrm{V_{Cd}^{-2}}$	0.70
$\mathrm{Se_{i}^{+}} + e_{\mathrm{CBM}}^{-} \rightarrow \mathrm{Se_{i}^{0}}$	0.71
$\mathrm{Se_{Cd}^{+}} + e_{\mathrm{CBM}}^{-} \rightarrow \mathrm{Se_{Cd}^{0}}$	0.57

5. Urbach Energy Extraction

The Urbach energy E_U was determined by fitting the exponential band-tail region of the absorbance according to

$$A(E) = A_0 \exp\left[\frac{E - E_0}{E_U}\right]$$

where E is the photon energy and E_U characterizes the width of the exponential tail. Practically, E_U was extracted from the inverse slope of a linear fit to $\ln(A)$ versus E over the sub-band-gap exponential region, where reflectance and interference effects are minimal. This approach is widely used to quantify band-edge disorder and tail states in semiconductors [19].

6. Absorption Spectra

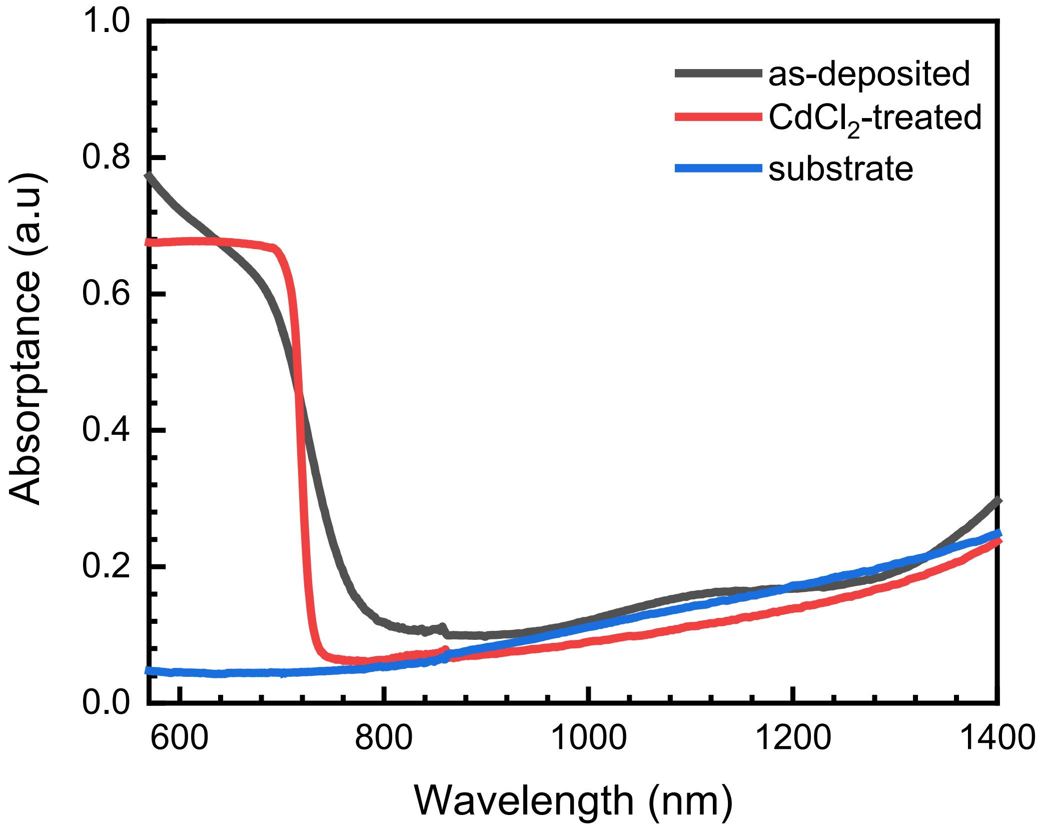

Supplementary Fig. 15. Absorptance spectra of as-deposited CdSe, $CdCl_2$-treated CdSe, and the bare substrate.

7. Supplementary References

[1]. E. Bastola *et al.*, "Cadmium Selenide (CdSe) as an Active Absorber Layer for Solar Cells with Voc Approaching 750 mV," in *2023 IEEE 50th Photovoltaic Specialists Conference (PVSC)*, 2023: IEEE, pp. 1-6.

[2]. Abudulimu, Abasi, et al. "Comprehensive Study of Carrier Recombination in High-Efficiency CdTe Solar Cells Using Transient Photovoltage." Solar RRL 8.10 (2024): 2400131.

[3]. Abudulimu, Abasi, et al. "Enhanced understanding of recombination mechanisms in high-performance tin-lead perovskite solar cells." Cell Reports Physical Science 6.1 (2025).

[4]. Abudulimu, Abasi, et al. "Crucial role of charge transporting layers on ion migration in perovskite solar cells." *Journal of Energy chemistry* 47 (2020): 132-137.

[5]. Abudulimu, Abasi, et al. "Photophysical properties of CdSe/CdTe bilayer solar cells: a confocal raman and photoluminescence microscopy study." *2022 IEEE 49th Photovoltaics Specialists Conference (PVSC)*. IEEE, 2022.

[6]. Kresse, Georg, and Jürgen Hafner. "Ab initio molecular dynamics for liquid metals." *Physical review B* 47.1 (1993): 558.

[7]. Kresse, Georg, and Jürgen Hafner. "Ab initio molecular-dynamics simulation of the liquid-metal–amorphous-semiconductor transition in germanium." *Physical Review B* 49.20 (1994): 14251.

[8]. Kresse, Georg, and Jürgen Furthmüller. "Efficient iterative schemes for ab initio total-energy calculations using a plane-wave basis set." *Physical review B* 54.16 (1996): 11169.

[9]. Blöchl, Peter E. "Projector augmented-wave method." *Physical review B* 50.24 (1994): 17953.

[10]. Heyd, Jochen, Gustavo E. Scuseria, and Matthias Ernzerhof. "Hybrid functionals based on a screened Coulomb potential." *The Journal of chemical physics* 118.18 (2003): 8207-8215.

[11]. Krukau, Aliaksandr V., et al. "Influence of the exchange screening parameter on the performance of screened hybrid functionals." *The Journal of chemical physics* 125.22 (2006).

[12]. Perdew, John P., Kieron Burke, and Matthias Ernzerhof. "Generalized gradient approximation made simple." *Physical review letters* 77.18 (1996): 3865.

[13]. Freysoldt, Christoph, Jörg Neugebauer, and Chris G. Van de Walle. "Fully ab initio finite-size corrections for charged-defect supercell calculations." *Physical review letters* 102.1 (2009): 016402.

[14]. Kumagai, Yu, and Fumiyasu Oba. "Electrostatics-based finite-size corrections for first-principles point defect calculations." *Physical Review B* 89.19 (2014): 195205.

[15]. Gake, Tomoya, et al. "Finite-size corrections for defect-involving vertical transitions in supercell calculations." *Physical Review B* 101.2 (2020): 020102.

[16]. Lany, Stephan, and Alex Zunger. "Accurate prediction of defect properties in density functional supercell calculations." *Modelling and simulation in materials science and engineering* 17.8 (2009): 084002.

[17]. Kavanagh, Seán R., et al. "doped: Python toolkit for robust and repeatable charged defect supercell calculations." *Journal of Open Source Software* 9.96 (2024).

[18]. Kumagai, Yu, et al. "Insights into oxygen vacancies from high-throughput first-principles calculations." *Physical Review Materials* 5.12 (2021): 123803.

[19]. Pankove, J. I. *"Optical Processes in Semiconductors." Prentice-Hall: Englewood Cliffs, NJ* (1971): pp. 36–38.
